# The influence of plasma sPD-L1 concentration on the effectiveness of immunotherapy in advanced NSCLC patients

**DOI:** 10.1007/s00262-023-03552-x

**Published:** 2023-10-10

**Authors:** Izabela Chmielewska, Anna Grenda, Paweł Krawczyk, Małgorzata Frąk, Barbara Kuźnar Kamińska, Weronika Mitura, Janusz Milanowski

**Affiliations:** 1 Department of Pneumonology, Oncology and Allergology Medical, University of Lublin, Jaczewskiego 8, 20-954 Lublin, Poland; 2 Department of Pulmonology, Allergology and Pulmonary Oncology, Poznan University of Medical Sciences, Poznań, Poland

**Keywords:** Non-small cell lung cancer, Immunotherapy, PD-L1, Soluble PD-L1

## Abstract

**Introduction:**

PD-L1 (Programmed Cell Death Ligand 1) is currently the only recognised marker of response to immunotherapy with anti-PD-1 or anti-PD-L1 antibodies in patients with advanced non-small cell lung cancer (NSCLC). However, this marker is not perfect. Soluble PD-L1 (sPD-L1) may be a novel predictor of immunotherapy efficacy in NSCLC patients.

**Material and methods:**

We enrolled 120 patients (median age 68 ± 6.81 years, 70 males and 50 females) with locally advanced (stage IIIB; 10 patients) or advanced (stage IV; 110 patients) NSCLC. PD-L1 expression in tumour cells was assessed by immunohistochemistry (IHC) in 117 (97.5%) patients. The soluble PD-L1 concentration in plasma samples was measured using enzyme-linked immunosorbent assay (ELISA). The response to immunotherapy, progression-free survival (PFS), and overall survival (OS), calculated from the start of immunotherapy, were assessed in 119 patients.

**Results:**

Patients with disease control had significantly lower (*p* = 0.0006) concentrations of sPD-L1 in blood plasma than patients with progression during the first months of immunotherapy or chemoimmunotherapy Patients with ≥ 6 month progression-free survival had a significantly higher (*p* = 0.013) percentage of tumor cells with PD-L1 expression than patients with shorter PFS. Patients with ≥ 6 months OS had significantly lower (*p* = 0.0142) plasma sPD-L1 concentrations than those with shorter overall survival. The median PFS was significantly higher in patients with low sPD-L1 concentrations than in those with high concentrations of this protein (5.8 vs. 2.5 months, HR = 0.6021, *p* = 0.0156). Similarly, patients with low sPD-L1 levels had a significantly higher median overall survival than those with sPD-L1 levels above the median (16.5 vs. 7 months, HR = 0.5354, *p* = 0.0071). There was no significant correlation between the percentage of tumour cells expressing PD-L1 and the concentration of sPD-L1 in the blood plasma.

**Conclusion:**

High sPD-L1 concentration is a negative predictor of immunotherapy efficacy in patients with NSCLC. It is worthwhile to determine sPD-L1 concentration to predict the risk of resistance to anti-PD-1 or anti-PD-L1 antibodies with greater certainty.

## Introduction

In patients with lung cancer, the only validated predictive marker qualifying for immunotherapy is the percentage of tumour cells (TC) expression of PD-L1 (Programmed Death Ligand 1) [1, 2]. The standard method for determining this expression is immunohistochemistry (IHC), which involves staining of tumour cells with anti-PD-L1 antibodies on microscopic slides. Immunotherapy monotherapy (atezolizumab, pembrolizumab, or cemiplimab) is used as the first-line treatment in advanced non-small cell lung cancer (NSCLC) patients with PD-L1 expression ≥ 50% of TC [3–5]. However, the combination of immunotherapy and chemotherapy should be considered as first-line therapy in patients with PD-L1 expression of < 50% of TC [6]. As a second-line treatment, immunotherapy can be used in patients with locally advanced or advanced NSCLC regardless of PD-L1 expression (nivolumab or atezolizumab) or in patients with PD-L1 expression on ≥ 1% of TC (pembrolizumab) [1, 7].

In both first- and second-line therapies, there is a risk of lack of a durable response and resistance to immunotherapy in many NSCLC patients [8, 9]. Even in some NSCLC patients with a high percentage of TC PD-L1 expression, treatment with anti-PD-1 or anti-PD-L1 antibodies may be ineffective. In contrast, immunotherapy can be beneficial in patients with low or no expression of this protein on TC [1, 10]. Therefore, additional markers would support the prediction of immunotherapy with Immune Checkpoint Inhibitors (ICIs) in NSCLC patients. One such predictor is the soluble form of PD-L1 (sPD-L1). The concentration of sPD-L1 in blood serum or plasma can be measured repeatedly in a non-invasive test with enzyme-linked immunosorbent assay (ELISA). In this approach, the material does not have to be fixed, as in the case of cancerous tissues or cells. Formali-fixed paraffin-embedded (FFPE) materials are subjected to chemical and thermal modifications that affect the specimen quality. Furthermore, unique materials containing tumour cells or tissues obtained by fine-needle aspiration (FNA) biopsy during bronchoscopy are often very sparse. Therefore, IHC staining results do not reflect PD-L1 expression in the entire tumour. Most often, we underestimate PD-L1 expression, which results in surprisingly high effectiveness of immunotherapy. However, fine-needle aspiration biopsy could reveal a tumour fragment with high PD-L1 expression, while the remaining tumour tissue had low PD-L1 expression. Thus, the effects of immunotherapy may be worse than expected. Moreover, the sPD-L1 concentration can be evaluated when it is impossible to collect cancer cells or tissues by FNA biopsy for PD-L1 expression assessment.

Studies have indicated that high expression of sPD-L1 in blood plasma or serum is a negative predictive factor for immunotherapy in various types of cancer [11]. There are indications that high concentrations of sPD-L1, together with high expression of ADAM10 (ADAM Metallopeptidase Domain 10) and ADAM17 (ADAM Metallopeptidase Domain 17) acted as predictors of poor response to immunotherapy in cancer patients [12].

We assessed sPD-L1 concentration in patients with advanced NSCLC treated with immunotherapy or chemoimmunotherapy as the first or second line of treatment. We considered whether the sPD-L1 concentration could be used as a predictive factor for patients treated with ICIs.

## Materials and methods

### Characteristics of the studied group

A two-centre, retrospective, non-randomised study enrolled 120 patients (median age 68 ± 6.81 years, 70 males and 50 females) with locally advanced (stage IIIB, 10 patients) or advanced (stage IV, 110 patients) NSCLC. The study group consisted of 61 patients with adenocarcinoma (50.8%), 50 patients with squamous cell carcinoma (SCC) (41.7%), seven patients with NSCLC NOS (5.8%), and two patients with large-cell neuroendocrine carcinoma (1.7%). The presence of mutations in the *EGFR* gene and rearrangement of the *ALK* and *ROS1* genes was excluded before qualification for treatment in non-SCC patients. PD-L1 expression was evaluated in 117 patients (97.5%). 95 patients (81.2%) showed PD-L1 expression in ≥ 1% of tumour cells, and 55 patients (47%)- on ≥ 50% of tumour cells. 51 patients (42.5%) with high PD-L1 expression received pembrolizumab as the first-line treatment. Twenty-two patients (18.3%) with PD-L1 expression on < 50% of TC patients received chemotherapy combined with pembrolizumab. 47 patients (39.2%) received second-line immunotherapy (nivolumab or atezolizumab), regardless of PD-L1 expression status. Responses to immunotherapy, progression-free survival (PFS), and overall survival (OS), calculated from the start of therapy, were assessed in 119 patients. The percentage of patients with 6-month and 6-month overall survival was also determined. 102 patients (85%) discontinued immunotherapy because of treatment progression or toxicity, and 80 deaths (66.7%) were reported at the end of follow-up. Patient characteristics are presented in Table 1.

**Table 1:** Characteristics of patients and assessment of progression-free survival and overall survival in association with clinical characteristics of patients, PD-L1 expression on tumor cells and blood plasma sPD-L1 concentration (*PFS and OS were calculated in 119 patients). Statistically significant results are in bold

		Number of patients n (%)	Median PFS months (95% CI)*	HR (95% CI), p	Median OS months (95% CI)*	HR (95% CI), p
Total		120 (100)	5 (3–5.8)	-	12.5 (8.5–16.5)	
Gender	Female	50 (41.7)	5 (3–8)	0.8677 (0.5765–1.3059), 0.4963	13 (9.5–23.8)	0.7754 (0.4924–1.2211), 0.2722
	Male	70 (58.3)	4.3 (3–5.8)		8.5 (7–16)	
Age	≥ 65 years	79 (65.8)	5 (3–7)	0.91 (0.5965–1.3885), 0.6619	12 (7.5–16)	1.3595 (0.8551–12.1614), 0.1942
	< 65 years	41 (34.2)	4.9 (2.5–6.5)		13.5 (7.5–27)	
Pathomorphological diagnosis	Squamous cell lung cancer	50 (41.7)	5 (3–7)	0.9134 (0.6098–1.3681), 0.6603	12.5 (8.5–18.5)	0.9188 (0.5852–1.4426), 0.713
	Non-squamous cell lung cancer	70 (58.3)	4.2 (3–6.5)		12 (7–18)	
Stage of disease	IIIB	10 (8.3)	5 (3–6.4)	0.9204 (0.4075–2.079), 0.8419	8.5 (4–16.5)	1.1729 (0.4789–2.873), 0.7271
	IV	110 (91.7)	2.8 (2–5.5)		12.5 (8–16.5)	
Line of immunotherapy	First line	73 (60.8))	5 (3–6.6)	1.0482 (0.6956–1.5795), 0.8219	9.5 (6.4–13.8)	1.2445 (0.7886–1.9642), 0.3473
	Second line	47 (39.2)	5 (3–6.5)		16 (9.5–18.5)	
sPD-L1 concentration	< 20 pg/mL	61 (50.8)	**5.8 (5–8.5)**	**0.6021 (0.3991–0.9084), 0.0156**	**16.5 (12–23.8)**	**0.5354 (0.3398–0.8435), 0.0071**
	≥ 20 pg/mL	59 (49.2)	**2.5 (2–4)**		**7 (4–13.5)**	
PD-L1 expression (PD-L1 expression was examined in 117 patients)	≥ 1% TC	95 (81.2)	5.4 (3–6.5)	0.6962 (0.3985–1.2166), 0.2036	12 (7.5–17)	0.8273 (0.4665–1.4674), 0.5168
	< 1% TC	22 (18.8)	3.5 (2.5–5.5)		12.5 (7–18)	
	≥ 50% TC	55 (47)	**6.5 (4–12)**	**0.584 (0.3865–0.8825), 0.0107**	13 (6.6–30.5)	0.6959 (0.4427–1.0937), 0.116
	< 50% TC	62 (53	**4 (2.6–5.5)**		11.5 (7–16)	

All the patients provided written informed consent to participate in the study. The study was approved by the local Bioethics Committee of the Medical University of Lublin (approval number – KE-0254/95/2018).

### Enzyme-linked immunosorbent assay (ELISA) for assessment of sPD-L1 concentration

Peripheral blood samples were collected before initiation of immunotherapy or chemotherapy. Blood samples were collected in tubes containing ethylenediaminetetraacetic acid (EDTA). The samples were then centrifuged for 10 min at 2,000 rpm. The obtained plasma was collected and stored at − 80 °C until the assay was performed. sPD-L1 concentration was determined using a Human PD-L1 ELISA Kit (Cat #BMS2327, ThermoFisher, Waltham, USA). The ELISA was performed according to the manufacturer’s instructions. Absorbance was measured at 450 nm using a BioTek ELx800 Absorbance Microplate Reader (BioTek, Winooski, VT, USA). A standard curve was generated, from which the concentrations were calculated. Analysis was performed using the Gen5 3.03 Microplate Reader and Imager Software (BioTek, Winooski, VT, USA).

### Statistical analysis

Data are expressed as numbers and percentages (for categorised variables) as well as medians and standard deviations (SD) (for continuous variables). We used the Mann–Whitney U test to examine the equality of population medians among groups with different demographic and clinical factors and plasma concentrations of sPD-L1. Spearman's correlation coefficient was used to measure the strength and direction of association between the two ranked variables. Kaplan–Meier analysis was used to compare the PFS and overall survival between the two groups. These tests were performed using Statistica v. 13.1 (Tibco Software, USA) and MedCalc (MedCalc Software Ltd, Ostend, Belgium) software. Statistical significance was set at *p* < 0.05.

## Results

### Immunotherapy or chemoimmunotherapy effectiveness in NSCLC patients

Partial remission (PR) occurred in 33 (27.5%) patients. Stable disease (SD) was found in 38 patients (31.7%), and progressive disease (PD) in 49 patients (40.8%). Disease control was achieved in 71 (59.2%) patients. 48 patients (40.3%) remained progression-free for ≥ 6 months. Survival longer than six months was observed in 80 patients (66.2%). The median PFS was 5 months (95% CI 3–5.8) and the median OS was 12.5 months (95% CI 8.5–16.5).

### PD-L1 expression on tumor cells and sPD-L1 concentration in plasma of NSCLC patients

The median and standard deviation of the tumour cell percentage with PD-L1 expression was 30 ± 33.12%, while the concentration of the soluble form of PD-L1 in the blood plasma was 19.8 ± 44.32 pg/mL. PD-L1 expression on tumour cells was similar in males and females (*p* = 0.329), and in older and younger patients (*p* = 0.818). The sPD-L1 concentration was insignificantly lower in women than in men (*p* = 0.078), and in younger patients than in older subjects (*p* = 0.123). Pathomorphological diagnosis (squamous cell carcinoma patients compared to non-squamous NSCLC patients) had no significant effect on the percentage of tumour cells expressing PD-L1 (*p* = 0.931) or on the concentration of sPD-L1 in the blood plasma (*p* = 0.678). The percentage of tumour cells expressing PD-L1 and the concentration of sPD-L1 were not significantly different between stage IIIB and IV patients (*p* = 0.244 and *p* = 0.447, respectively). There were no significant differences (*p* = 0.683) in sPD-L1 plasma concentrations between patients without PD-L1 expression and patients with PD-L1 expression on tumour cells (≥ 1% of TC). Patients with high PD-L1 expression (≥ 50% TC) showed a similar (*p* = 0.896) sPD-L1 concentration to those with PD-L1 expression on > 50% of the tumour cells. There was no significant correlation between the percentage of tumour cells expressing PD-L1 and the concentration of sPD-L1 in blood plasma (R =  + 0.0312, *p* = 0.738).

### Influence of PD-L1 expression on tumor cells and sPD-L1 concentration in blood plasma on the effectiveness of immunotherapy or chemoimmunotherapy

**Fig. 1:**
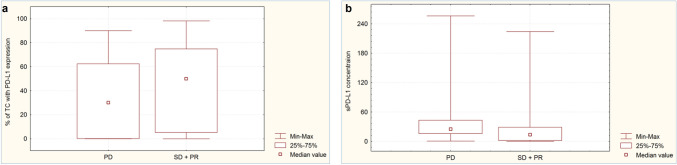
Percentage of tumor cells with PD-L1 expression (**a**) and concentration of plasma sPD-L1 (**b**) in patients with disease progression or control

**Fig. 2:**
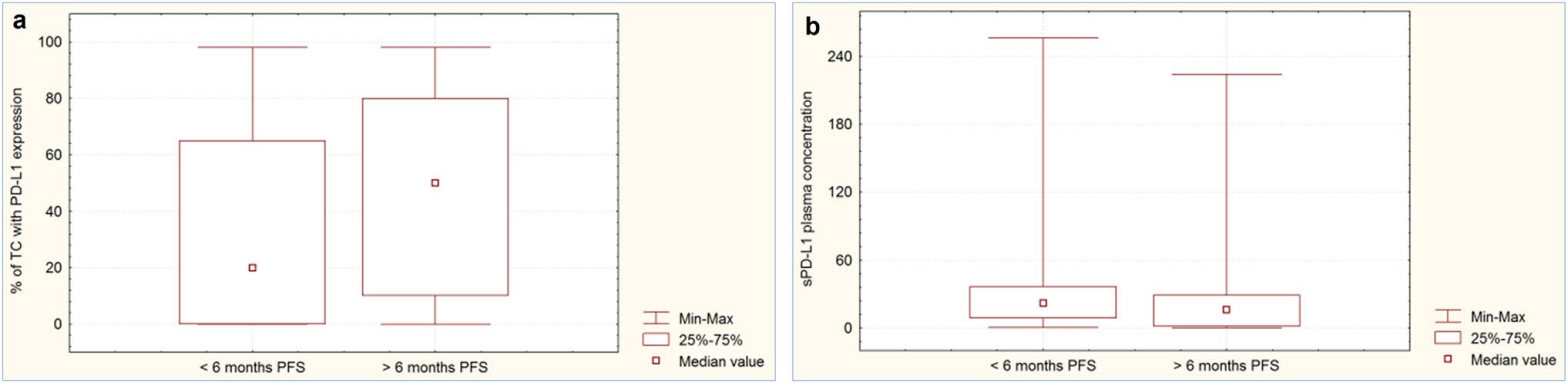
Percentage of tumor cells with PD-L1 expression (**a**) and concentration of plasma sPD-L1 (**b**) in patients with ≥ 6 or < 6 months progression-free survival

**Fig. 3:**
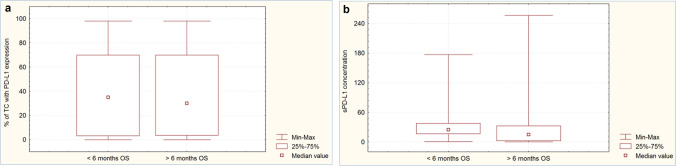
Percentage of tumor cells with PD-L1 expression (**a**) and concentration of plasma sPD-L1 (**b**) in patients with ≥ 6 or < 6 months overall survival

The percentage of tumour cells with PD-L1 expression was not significantly higher (*p* = 0.086) in patients with disease control at the first assessment during immunotherapy or chemoimmunotherapy than in those with disease progression (Fig. 1a). Patients with disease control had significantly lower concentrations of sPD-L1 in the blood plasma than patients with disease progression during the first months of immunotherapy or chemoimmunotherapy (13.72 pg/mL vs. 25.03 pg/mL, *p* = 0.0006, Fig. 1b). Patients with ≥ 6 months of PFS had a significantly higher (*p* = 0.013) percentage of tumour cells expressing PD-L1 (Fig. 2a) and a non-significantly lower sPD-L1 plasma concentration than patients with shorter PFS (16.34 pg/mL vs. 22.13 pg/mL, *p* = 0.0626, Fig. 2b). The percentage of tumor cells withPD-L1 expression was similar (*p* = 0.958) in patients with overall survival longer and shorter than six months (Fig. 3a). However, patients with ≥ 6 months OS had significantly lower plasma sPD-L1 concentrations than those with shorter overall survival (14.93 pg/mL vs. 25.03 pg/mL, *p* = 0.0142, Fig. 3b).

**Fig. 4:**
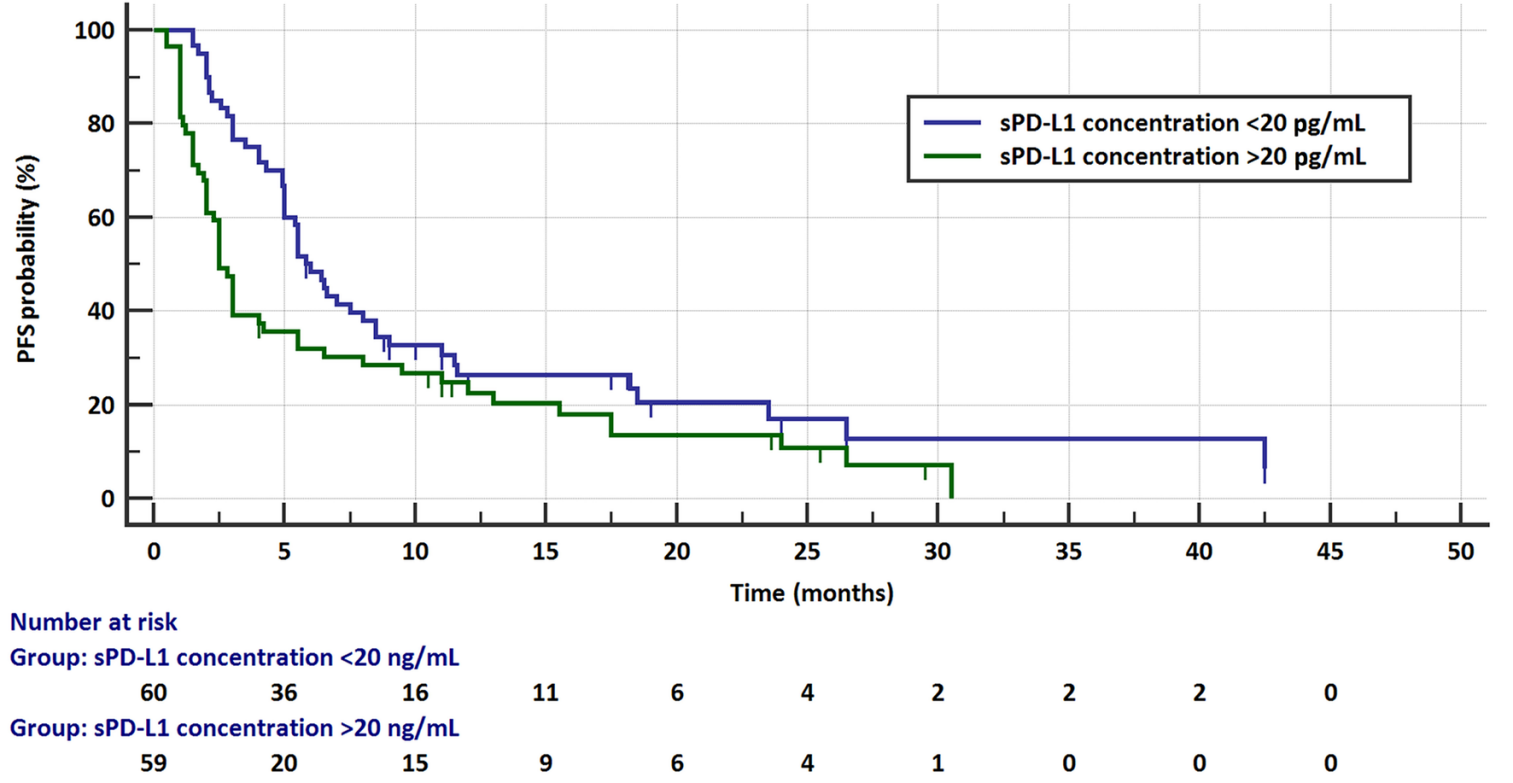
Progression-free survival in patients with low (< 20 pg/mL) and high (≥ 20 pg/mL) concentration of plasma sPD-L1

**Fig. 5:**
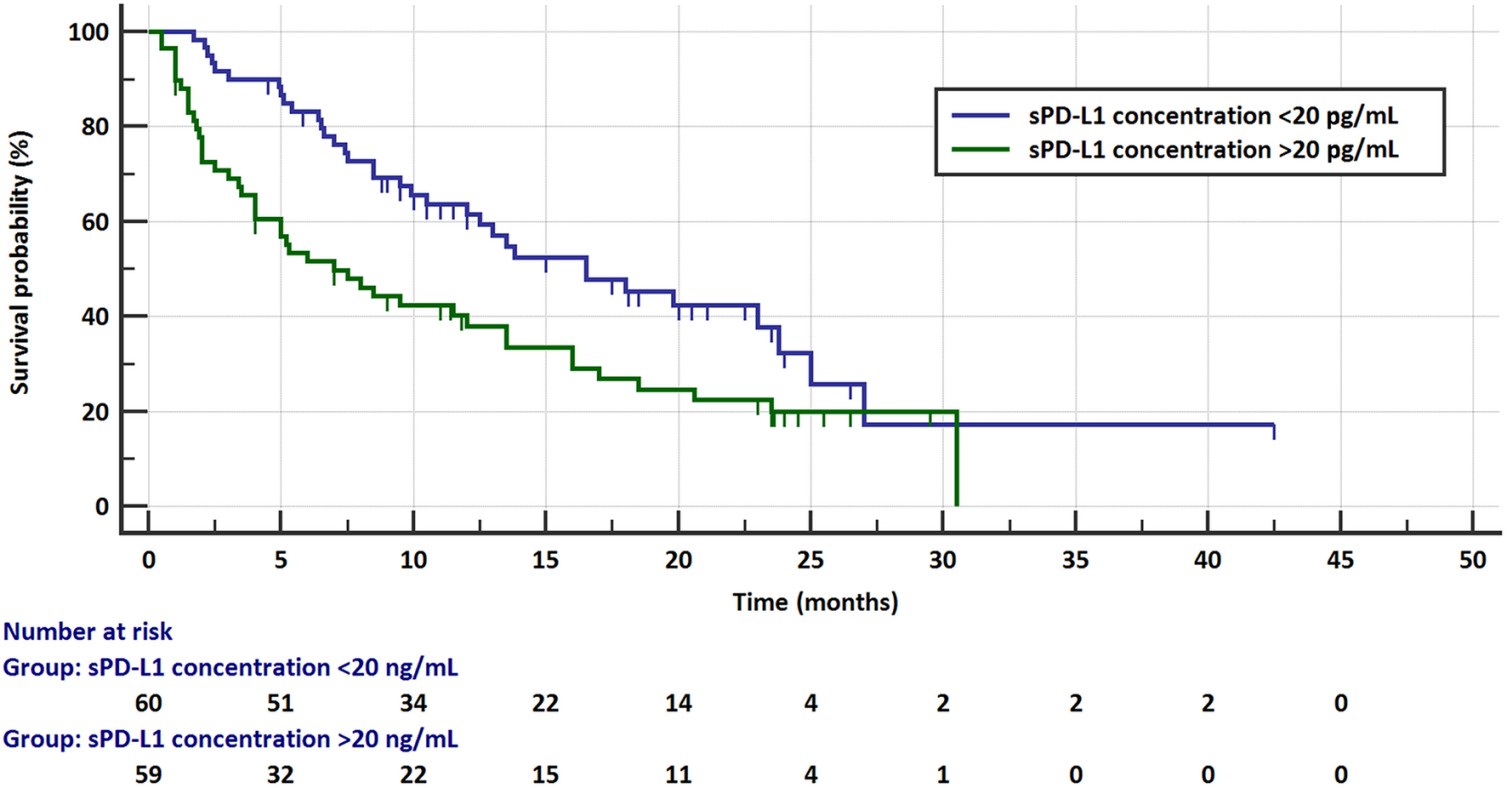
Overall survival in patients with low (< 20 pg/mL) and high (≥ 20 pg/mL) concentration of plasma sPD-L1

**Fig. 6:**
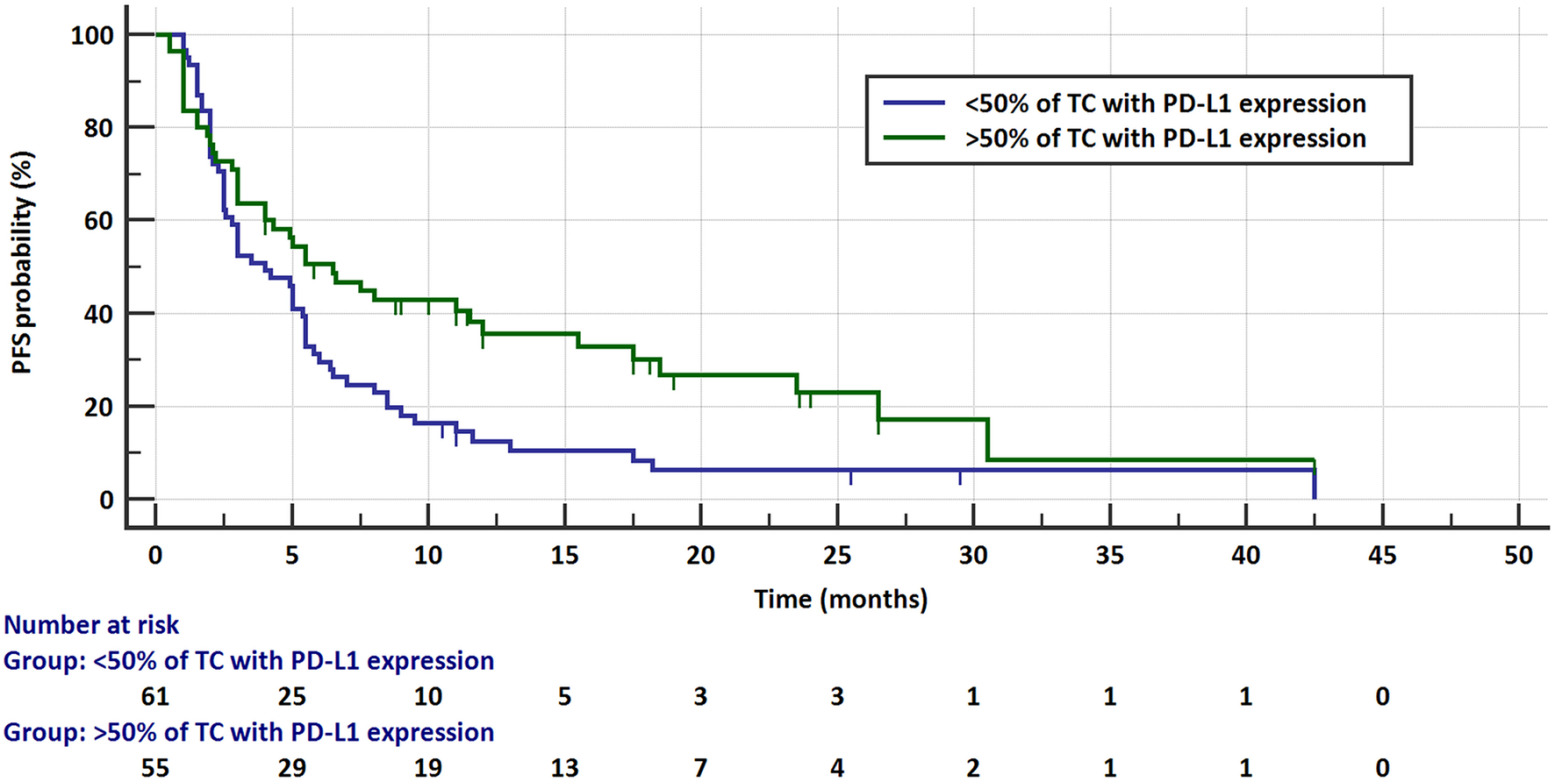
Progression-free survival in patients with expression of PD-L1 on ≥ 50% of TC and in patients with lower expression of PD-L1

The risk of progression and the risk of death did not depend on the age and sex of the patients or on the pathomorphological diagnosis, stage of disease, or the line of treatment in which immunotherapy was applied. The median PFS was significantly higher in patients with sPD-L1 plasma concentrations < 20 pg/mL (5.8 months, 95% CI 5–8.5 months) than in those with ≥ 20 pg/mL (2.5 months, 95% CI 2–4 months) concentrations of this protein (HR = 0.6021, 95% CI 0.3991–0.9084, *p* = 0.0156, Fig. 4). Similarly, patients with sPD-L1 levels < 20 pg/mL had a significantly higher median overall survival (16.5 months, 95% CI 12–13.8 months) than those with sPD-L1 (7 months, 95% CI 4–13.5 months) above this value (HR = 0.5354, 95% CI 0.3398–0.8435, *p* = 0.0071, Fig. 5). The risk of progression was also lower in patients with PD-L1 expression on ≥ 50% of TC (6.5 months, 95% CI 4–12 months) than in patients (4 months, 95% CI 2.6–5.5) with lower PD-L1 expression (HR = 0.584, 95% CI 0.3865–0.8825, *p* = 0.0107, Fig. 6). However, there were no significant differences in the risk of death between the groups with different PD-L1 progression on TC. Patients with PD-L1 expression on ≥ 1% TC and < 1% TC had a comparable risk of progression and death. Table 1 presents the results.

## Discussion

High concentrations of plasma sPD-L1 have been found to be a negative predictive factor for immunotherapy efficacy in patients with advanced NSCLC. Furthermore, there was no correlation between plasma sPD-L1 levels and PD-L1 expression on TC. This may be due to the widespread expression of PD-L1 on normal cells, including very high expression of on antigen-presenting cells (dendritic cells, macrophages, and B lymphocytes) or activated T lymphocytes [13]. Oh et al. suggested that sPD-L1 in patients with advanced cancer is mainly derived from neutrophils [14]. In a study by Donahue et al., 5–35% of peripheral blood myeloid-derived suppressor cells (MDSCs) expressed PD-L1 in cancer patients [15]. The expression of PD-L1 was highest in granulocytic myeloid-derived suppressor cells and lowest in T, NK, and B cells [15].

Castello et al. suggested that the increase in sPD-L1 concentration during ICIs treatment may reflect the expansion of the tumor volume [16]. A meta-analysis by Scirocchi showed that high levels of sPD-L1 correlated with shorter OS and PFS in patients with different types of cancer treated with immunotherapy (HR = 1.49, 95% CI 1.15–1.93, *p* < 0.01; OS: HR = 1.59, 95% CI 1.20–2.12, *p* < 0.01, for PFS). In NSCLC patients, high levels of sPD-L1 were associated with a shortening of progression-free survival and overall survival (HR = 1.81, 95% CI 1.09–3.00, *p* = 0.02 OS; HR = 2.18, 95% CI 1.27–3.76, *p* < 0.01 for PFS) [11]. Scirocchi et al. suggested that high sPD-L1 levels in cancer patients indicate worse survival and may be a helpful biomarker for qualification for immunotherapy, thus improving the efficacy of ICIs and avoiding unnecessary treatment [11]. Zizzari et al. indicated that a low level of sPD-L1 is correlated with a long response to nivolumab treatment in patients treated with nivolumab [17]. Murakami et al. proved that in patients with non-small cell lung cancer treated in first, second or third-line with anti-PD-1 antibody, PFS and OS in the high sPD-L1 group were significantly shorter than those in the low sPD-L1 patients (median PFS was 1.9 vs. 5.9 months with *p* = 0.011 and median OS was 6.1 months vs. not reached with *p* < 0.001) [18]. High serum sPD-L1 levels were independently associated with risk of progression (HR = 1.91, *p* = 0.061) or death (HR = 2.073, *p* = 0.034). The study of Okuma et al. on 96 patients with advanced lung cancer indicated that overall survival was significantly reduced in patients with high (> 7.32 ng/mL) concentration of plasma sPD-L1 compared with low (< 7.32 ng/mL) sPD-L1 level (13.0 vs. 20.4 months, *p* = 0.037). Furthermore, in multivariate analysis, high sPD-L1 levels were significantly associated with poor prognosis (HR = 1.99, *p* = 0.041) [19]. In a study by Oh et al., blood samples were obtained before and after ICIs treatment in 128 patients with advanced malignancies (inter alia melanoma, lung, and urothelial cancers), and an increase in sPD-L1 levels was detected after immunotherapy. They showed that patients with a high level (> 11.0 pg/μL) of sPD-L1 were more likely to exhibit progressive disease than those with a low level (41.8% vs. 20.7%, *p* = 0.013) [14]. The authors indicated that the median PFS was 2.9 months for patients with high level of sPD-L1 (95% CI 2.1–3.7 months) compared to 6.3 months in the patients with low concentration of this protein (95% CI 3.0–9.6 months, *p* = 0.023) [14]. The median OS was 7.4 months (95% CI 6.3–8.5 months,) versus 13.3 months (95% CI 9.2–17.4 months, *p* = 0.005) in these groups, respectively. The authors indicated that high sPD-L1 levels were an independent negative factor for both the risk of progression and death (hazard ratio [HR] = 1.928, *p* = 0.038 for PFS and HR = 1.788, *p* = 0.004 for OS) [14]. Similar to the results cited above, in our study, we showed that the median PFS and OS were significantly lower in patients with high levels of sPD-L1 than in those with low concentrations of this protein and that patients with high concentrations of plasma sPD-L1 had a higher risk of progression and death.

Furthermore, sPD-L1 levels did not correlate with tissue PD-L1 expression which was consistent with the results of our study [14]. In contrast, a statistically significant correlation between serum sPD-L1 concentration and tumour PD-L1 expression (R =  + 0.214, *p* = 0.001) was observed by Murakami et al. [18] Frigola et al. detected that sPD-L1 retains its receptor-binding domain, which could deliver pro-apoptotic signals to T lymphocytes [20]. Moreover, a higher preoperative level of this protein in patients with clear cell renal carcinoma is associated with larger tumours, higher advanced stage, higher grade, and area of necrosis. High sPD-L1 levels are also associated with an increased risk of death [20]. The authors indicated that sPD-L1 may promote tumour progression and subsequent poor clinical outcomes. Castello et al. found a significant association between a high level of sPD-L1 (above the median value of 27.22 pg/mL) and a high metabolic tumour burden expressed by metabolic tumour volume (*p* = 0.034) and total lesion glycolysis (*p* = 0.049) [16].

It should be mentioned that different ELISAs have different performances and that no sPD-L1 ELISA has certification for clinical. The tests may have different sensitivity and detection ranges. Nevertheless, it would be advisable to carry out inter-center validation for the selection of the optimal assay range with the aim of introducing a certified ELISA for the determination of sPD-L1 in clinical practice in terms of immunotherapy.

The mechanism by which sPD-L1 reduces the effectiveness of anti-PD-L1 or anti-PD-1 antibodies is different, but relatively simple to explain. Anti-PD-L1 antibodies bind to sPD-L1 instead of membrane PD-L1 on tumour cells. This reduces drug availability and leaves PD-L1 on tumour cells capable of inhibiting T-cell function. However, the circulating form of PD-L1 may compete with anti-PD-1 antibodies for binding to the cellular form of PD-1 on T cells; therefore, treatment may be ineffective. Moreover, sPD-L1 may act as a PD-1 agonist (as opposed to antagonist anti-PD-1 antibodies), which enhances T cell anergy.

Gong et al. showed that sPD-L1 expression may be associated with the emergence of resistance to immunotherapy. The authors identified two unique secreted PD-L1 splicing variants that lacked the transmembrane domain in NSCLC patients resistant to anti-PD-L1 antibodies. These secreted PD-L1 variants function as decoys of anti-PD-L1 antibodies. The authors experimented with mouse tumour cell cultures in which anti-PD-1 antibody treatment overcame resistance mediated by secreted PD-L1 variants [21].

Orme et al. showed that high expression of ADAM10 and ADAM17, together with high concentrations of sPD-L1, acted as predictors of poor response to immunotherapy in cancer patients [12]. ADAM10 and ADAM17 cleave PD-L1 from the surface of tumour and respiratory tract cells. This leads to the release of an active form of sPD-L1 that is capable of binding to PD-1 and inducing apoptosis in CD8 + T cells. Therefore, the anticancer activity of cytotoxic T cells is impaired. The authors also indicated a correlation between high mRNA expression of metalloproteinase ADAM10/17 in tumour cells and the concentration of sPD-L1 in peripheral blood [12]. The authors concluded that metalloproteinases could release PD-L1 into the bloodstream, where sPD-L1 captures the administered immunotherapeutics; thus, the treatment could not be fully effective. In addition, the expression of ADAM10 and ADAM17 has an indirect effect on inducing anergy and death of CD8 + T cells, which is related to the effects on PD-1 positive lymphocytes of the soluble form of sPD-L1. However, whether the use of ADAM10 and ADAM17 in combination with PD-1 and PD-L1 inhibitors abolishes this immune response has not been determined. This would restore the possibility of immunotherapy in NSCLC patients with the expression of the discussed metalloproteinases in the case of progression during therapy with anti-PD-1 or anti-PD-L1 antibodies [12, 22]. There are also suggestions that therapeutic plasma exchange (TPE) may deplete extracellular forms of PD-L1 associated with ICI resistance [12, 23]. Therefore, Davidson et al. designed two-arm study with metastatic melanoma patients progressing on checkpoint inhibition with an indication that TPE may rescue and restore antimelanoma immunity [23]. The study is ongoing and a patients with baseline sPD-L1 level of ≥ 1.7 ng/mL and adequate clinical capacity will be enrolled to TPE [23]. Researchers are also examining the kinetics of the sPD-L1 content (to be removed by TPE). The estimated completion date of the study is the end of October 2024 (NCT04581382, clinicaltrials.gov).

## Conclusion

High sPD-L1 concentration was a negative predictive factor for immunotherapy efficacy in patients with NSCLC. It would be worthwhile to determine the sPD-L1 concentration before the start of immunotherapy to predict the risk of resistance to anti-PD-1 or anti-PD-L1 antibodies with greater certainty. The availability and reproducibility of sPD-L1 determination using ELISA in the peripheral blood of NSCLC patients support the performance of this assay.
